# Filter Paper Blood Spot Enzyme Linked Immunoassay for Insulin and Application in the Evaluation of Determinants of Child Insulin Resistance

**DOI:** 10.1371/journal.pone.0046752

**Published:** 2012-10-08

**Authors:** Richard M. Martin, Rita Patel, Alexander Zinovik, Michael S. Kramer, Emily Oken, Konstantin Vilchuck, Natalia Bogdanovich, Natalia Sergeichick, Robert Gunnarsson, Lisa Grufman, Ying Foo, Nina Gusina

**Affiliations:** 1 School of Social and Community Medicine, University of Bristol, Bristol, United Kingdom; 2 MRC Centre for Causal Analyses in Translational Epidemiology, School of Social and Community Medicine, University of Bristol, Bristol, United Kingdom; 3 National Research and Applied Medicine, Mother and Child Centre, Minsk, Republic of Belarus; 4 Department of Epidemiology, Biostatistics and Occupational Health, McGill University, Montreal, Canada; 5 Department of Pediatrics, McGill University, Montreal, Canada; 6 Department of Population Medicine, Harvard Medical School and Harvard Pilgrim Health Care Institute, Boston, Massachusetts, United States of America; 7 Mercodia AB, Uppsala, Sweden; Copenhagen University Hospital, Denmark

## Abstract

**Background:**

In large-scale epidemiology, bloodspot sampling by fingerstick onto filter paper has many advantages, including ease and low costs of collection, processing and transport. We describe the development of an enzyme-linked immunoassay (ELISA) for quantifying insulin from dried blood spots and demonstrate its application in a large trial.

**Methods:**

We adapted an existing commercial kit (*Mercodia Human Insulin ELISA, 10-1113-01*) to quantify insulin from two 3-mm diameter discs (≈6 µL of blood) punched from whole blood standards and from trial samples. Paediatricians collected dried blood spots in a follow-up of 13,879 fasted children aged 11.5 years (interquartile range 11.3–11.8 years) from 31 trial sites across Belarus. We quantified bloodspot insulin levels and examined their distribution by demography and anthropometry.

**Results:**

Mean intra-assay (n = 157) coefficients of variation were 15% and 6% for ‘low’ (6.7 mU/L) and ‘high’ (23.1 mU/L) values, respectively; the respective inter-assay values (n = 33) were 23% and 11%. The intraclass correlation coefficient between 50 paired whole bloodspot versus serum samples, collected simultaneously, was 0.90 (95% confidence interval 0.85 to 0.95). Bloodspot insulin was stable for at least 31 months at −80°C, for one week at +30°C and following four freeze-thaw cycles. Paediatricians collected a median of 8 blood spots from 13,487 (97%) children. The geometric mean insulin (log standard deviation) concentrations amongst 12,812 children were 3.0 mU/L (1.1) in boys and 4.0 mU/L (1.0) in girls and were positively associated with pubertal stage, measures of central and peripheral adiposity, height and fasting glucose.

**Conclusions:**

Our simple and convenient bloodspot assay is suitable for the measurement of insulin in very small volumes of blood collected on filter paper cards and can be applied to large-scale epidemiology studies of the early-life determinants of circulating insulin.

## Introduction

Raised insulin levels in children are a marker for insulin resistance, [1], [2] elevated cardiovascular disease risk factors[3]–[6] and early atherosclerosis. [7] Furthermore, high insulin levels in childhood track into adulthood, [8], [9] when they are associated with type 2 diabetes,[10]–[12] cardiovascular disease[13]–[16] and premature mortality. [16], [17] There is, therefore, substantial interest in large-scale epidemiology studies of the genetic and environmental determinants of insulin levels in childhood[18]–[27] to inform strategies for the prevention of insulin resistance and its sequelae. [28].

Important challenges in large-scale epidemiology include non-acceptance of venepuncture by children and/or their parents; the costs, safety and logistics of serum or plasma separation by centrifugation; and frozen storage and transport of aliquots. An alternative procedure involves capillary puncture of the finger pulp to collect bloodspots which are then dried on filter paper. [29], [30] The major advantages of dried blood spot sampling are minimal training, lower cost than venepuncture, acceptability to parents and children, [30] negligible processing requirements (cards must be air dried), low biohazard risk because samples cannot leak and the ease of storage and transport of filter paper cards. [29], [31].

The use of dried blood spots is currently limited to large-scale paediatric screening programs for rare inherited disorders. There is, however, interest in developing and validating a wide range of bloodspot assays, and developments include radioimmunoassay [32] and chemiluminescence [33] methods for insulin quantification in dried blood spots. Radioimmunoassays require regulatory compliance, because of the use of radioisotopes, and both radioimmunoassay and chemiluminescence methods require specialised and costly equipment (radiation-counting apparatus or a luminometer, respectively) that may not be present in many laboratories. We are not aware of any reports using enzyme-linked immunoassay (ELISA), which offers the important advantage that results can be read on universally available microtitre plate readers, without the need for regulatory approvals to use radioisotopes or more specialised and costly measuring equipment. Development and validation of an ELISA blood spot assay offers a simple, convenient and novel alternative method of measuring insulin in large-scale epidemiology studies in a wide range of settings.

We describe the development and validation of an ELISA for quantification of insulin from dried blood spots using a simple adaptation of an existing commercial kit originally designed for use on serum or plasma (*Mercodia Human Insulin ELISA, catalogue number: 10-1113-01, Mercodia AB, Sweden*). The kit quantifies insulin using a solid phase, two-site enzyme immunoassay, based on the direct sandwich technique in which two monoclonal antibodies are directed against separate antigenic determinants on the insulin molecule. We show the successful practical application of our dried blood spot assay in a large-scale, multicentre trial (the Promotion of Breastfeeding Intervention Trial, PROBIT [34]) by describing the distribution and correlates of fasting insulin levels measured on over 12,000 children aged 11.5 years from 31 polyclinics distributed across the Republic of Belarus.

**Figure 1:**
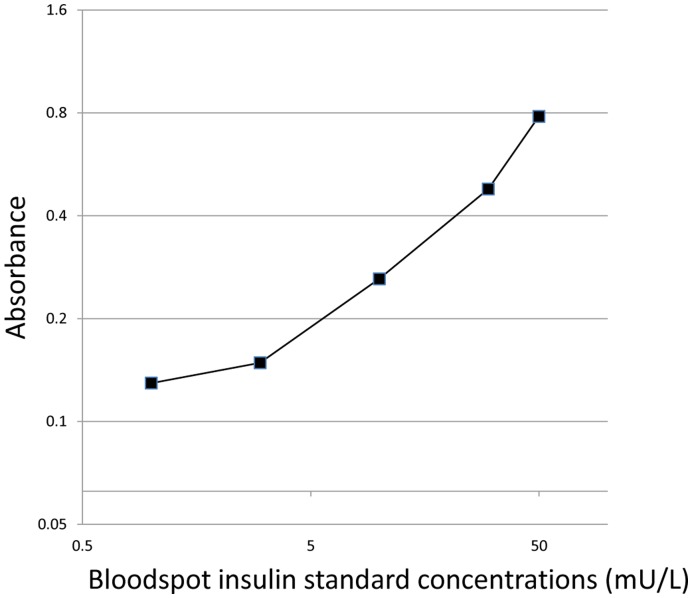
Typical calibration curve. Whole bloodspot insulin standards at 0, 1, 3, 10, 30 and 50 mU/L (log-log scale).

**Table 1:** Intra- and inter-assay imprecision of dried whole bloodspot insulin assays.

Sample	Mean insulin (mU/L)	Mean standard deviation of repeatedmeasures	Coefficient of variation
*Intra-assay (n = 157)*			
1	6.7	1.02	15%
2	23.1	1.35	6%
*Inter-assay (n = 33)*			
1	6.7	1.55	23%
2	23.1	2.57	11%

## Materials and Methods

### Materials

Blood samples were collected onto Food and Drug Administration approved Whatman 903 filter paper cards, [35] pre-printed with eight 10 mm diameter circles, standardized to absorb blood in a homogeneous manner so that uniform punches from any section of the sample yield the same quantity of blood. [36] The Human Insulin ELISA kit was from *Mercodia AB*, Sweden, which has no detectable cross-reactivity against c-peptide or proinsulin (both <0.01%) and showed excellent agreement against an isotope dilution–liquid chromatography/tandem mass spectrometry (IDMS) measurement procedure calibrated using purified recombinant insulin. [37] This kit was modified by *Mercodia*, in collaboration with the PROBIT study investigators, to produce a *Mercodia* Blood Spot Insulin ELISA kit, using the same antibodies as in the Human Insulin ELISA kit (*catalogue number: 10-1113-01*). Instruments used included an automatic filter paper card puncher (Wallac DBS (Dried Blood Spot) Puncher, product number: 1296-071, Perkin Elmer, USA), a microplate washing device (DELFIA Washer-Diskremove, product number: 1296-0010 Perkin Elmer, USA), a plate shaker (DELFIA Plateshaker, product number:1296-003, Perkin Elmer, USA) and a microplate reader (VICTOR3 Multilabel Reader, product number: 1420-012, Perkin Elmer, USA) with a 450 nm filter.

**Figure 2:**
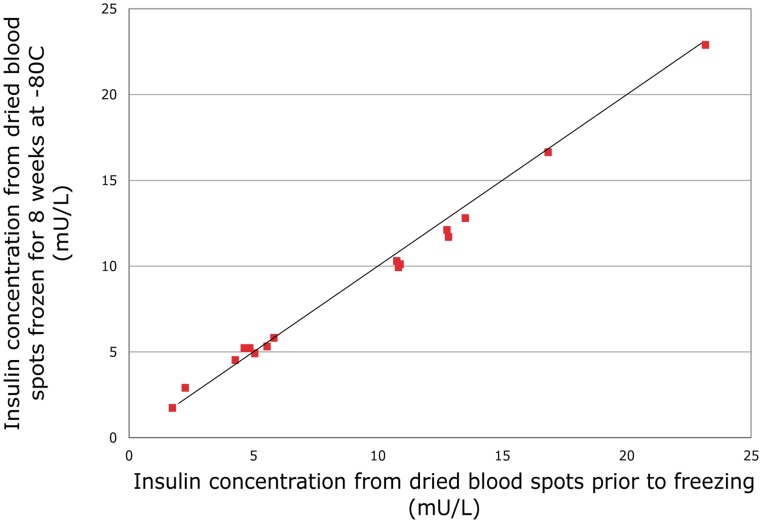
Stability of insulin concentrations measured from dried blood spots at −80°C for 8 weeks. Comparison of fasting insulin values for dried blood spot samples stored at **−**80°C for 8 weeks versus fasting insulin concentrations measured from dried blood spots prior to storage.

**Figure 3:**
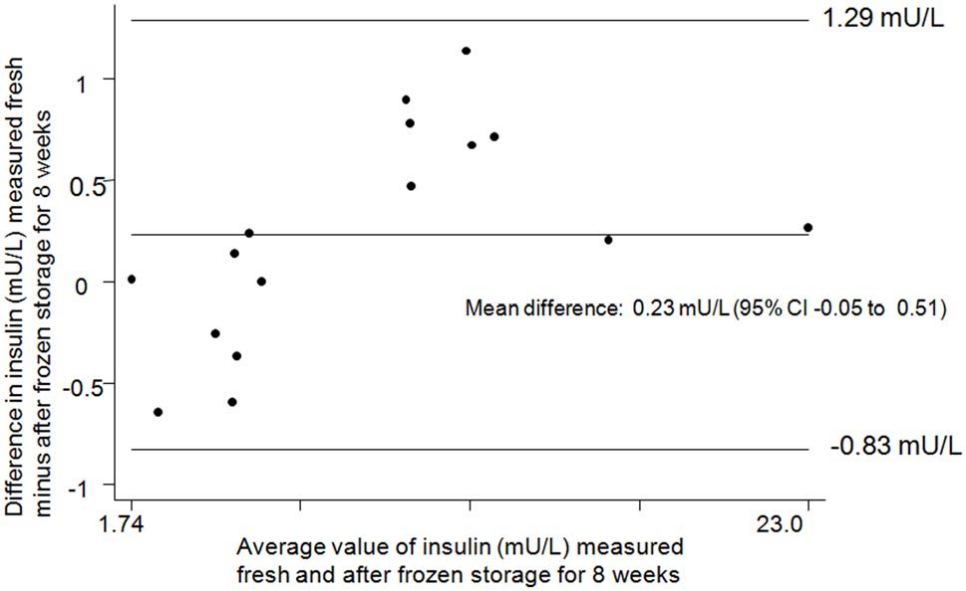
Bland-Altman plot for insulin values from fresh minus frozen samples. The difference in fasted bloodspot insulin from fresh sample minus the same sample frozen for 8 weeks at −80°C.

**Figure 4:**
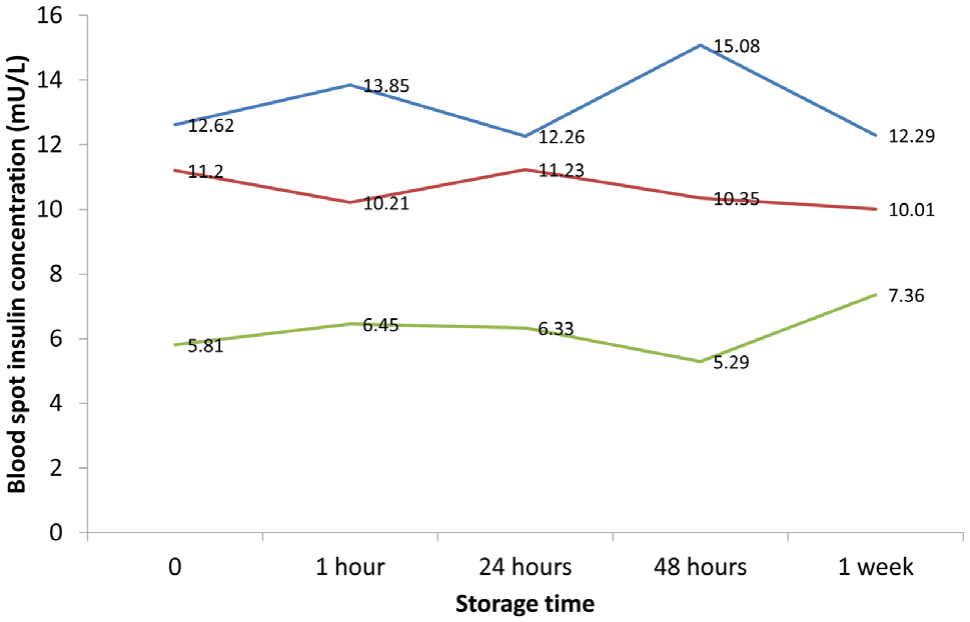
Stability of insulin concentrations measured from dried blood spots at +30°C for 1 weeks. Bloodspot fasting insulin concentrations from three samples analysed at baseline (time = 0), and after 1 hour, 12 hours, 24 hours, 48 hours and 1 week at +30°C.

### Preparation of Bloodspot Insulin Standards and Internal Quality Control Samples

To minimize matrix differences and maximize comparability between the standards, quality control samples and the trial samples, whole blood insulin standards (diluted to 1, 3, 10, 30 and 50 mU/L whole blood) and internal quality control samples were prepared by Mercodia using recombinant human insulin in insulin ELISA calibrator buffer, article number 20-2615, mixed with washed red blood cells (in proportions 60∶40 for a 0.40 haematocrit), followed by application to Whatman 903 filter paper. Standards were calibrated against the Human Insulin ELISA kit, in turn calibrated against the 1st International Reference Preparation 66/304. The detection limit (1mU/L) in dried blood spots was determined using the methodology described in ISO11843-part 4. [38].

**Figure 5:**
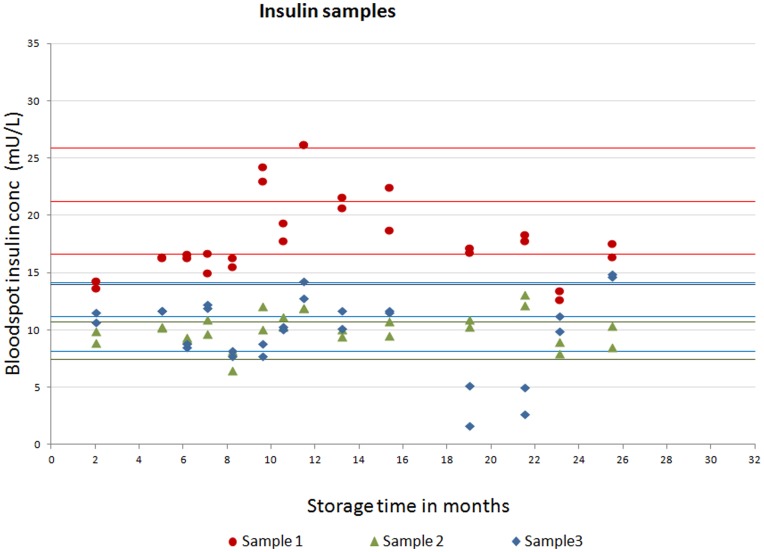
Stability of fasting insulin stored at −80°C for 26 months. Fasting insulin concentrations of three bloodspot samples analysed prior to freezing (time 0) and then at regular intervals over 26 months. For sample 1, the middle red line is the insulin concentration at time 0 and the upper and lower red lines are the 95% reference range (calculated from the standard deviation of 30 replicates of the time 0 value). For samples 2 and 3, the lines are coloured green and blue, respectively.

**Figure 6:**
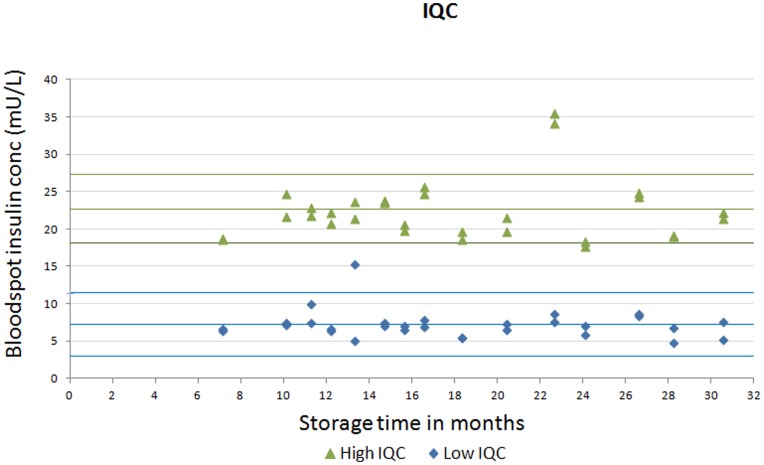
Stability of internal quality bloodspot samples stored at−80°C for 31 months. Fasting insulin concentrations of internal quality control (IQC) bloodspots analysed at regular intervals over 31 months. For the ‘low’ IQC sample, the middle blue line is the mean insulin concentration and the upper and lower blue lines are the 95% reference range (calculated from the standard deviation of 40 replicates); the respective lines for the ‘high’ IQC sample are coloured green.

**Figure 7:**
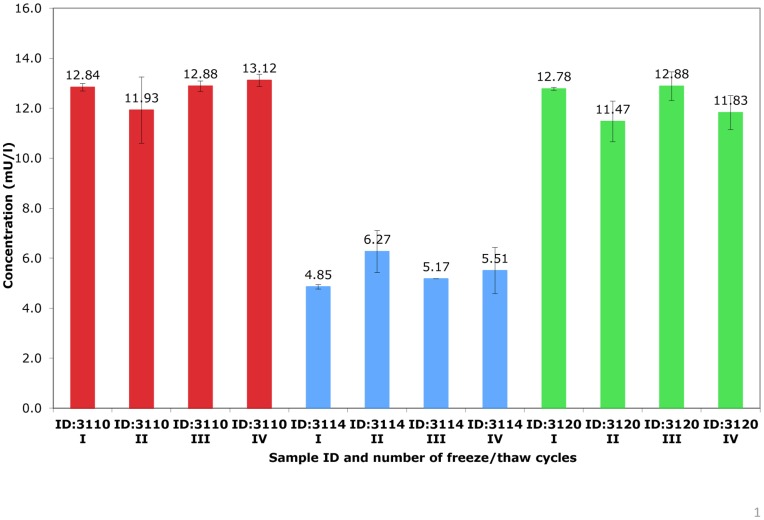
Fasting insulin concentrations by number of freeze-thaw cycles.

### Assay Procedure

All frozen blood spot cards (see sample collection below) were brought to room temperature before being removed from the plastic bags for assaying. Two 3-mm discs (≈ 6 µL of blood) were punched from the blood spots (of the standards, quality control samples and the trial samples) using an automatic puncher into 96-well microtitre plates, precoated with mouse anti-human insulin monoclonals. Two 3-mm discs were used in each assay well to ensure an adequate sample volume and elution from the bloodspots. Insulin was extracted from the discs by incubation for at least 16 hours in a refrigerator with 100 µL peroxidase conjugated mouse monoclonal anti-insulin diluted with Mercodia enzyme conjugate buffer, article number 20-2627. Following incubation, the contents of the wells were discarded, any remaining filter paper discs carefully removed and the plates washed 6 times with 350 µL of wash solution (Tris-buffered saline) to remove unbound enzyme labelled antibody. The bound conjugate was detected by a 30 minute incubation with 200 µL of 3,3′,5,5′-tetramethylbenzidine (TMB) added to each well before adding 50 µL of stop solution of 0.5 mol/L of H_2_SO_4_ to terminate the peroxidase/TMB reaction and give a colorimetric endpoint that was read spectrophotometrically at 450 nm. The run-specific absorbance of the standards was plotted against the assigned concentration in mU/L of the whole blood filter paper standard values (omitting calibrator 0) using the log scale on both x and y axes. The concentration of the samples was read from the smoothed cubic spline fitted standard curve. Samples with insulin levels above the highest standard (50 mU/L) were reanalyzed by using only one 3-mm disc instead of two (effectively diluting 1 in 2) and the results multiplied by 2.

**Figure 8:**
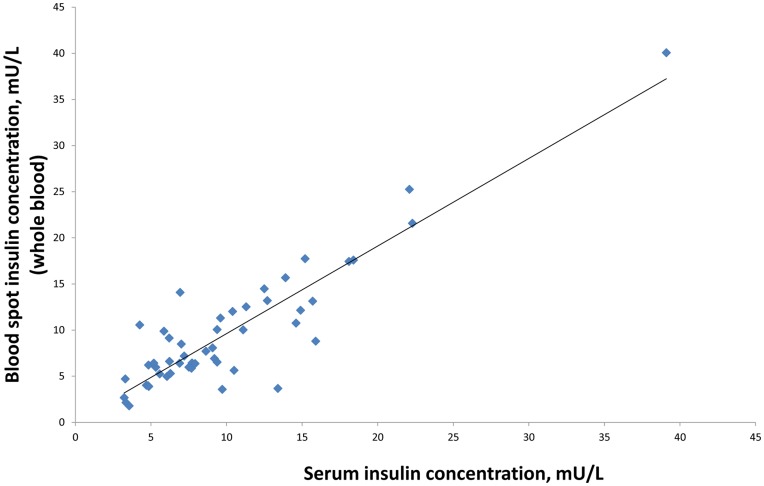
Comparison of fasting insulin measured from 50 paired serum and whole bloodspot samples.

**Figure 9:**
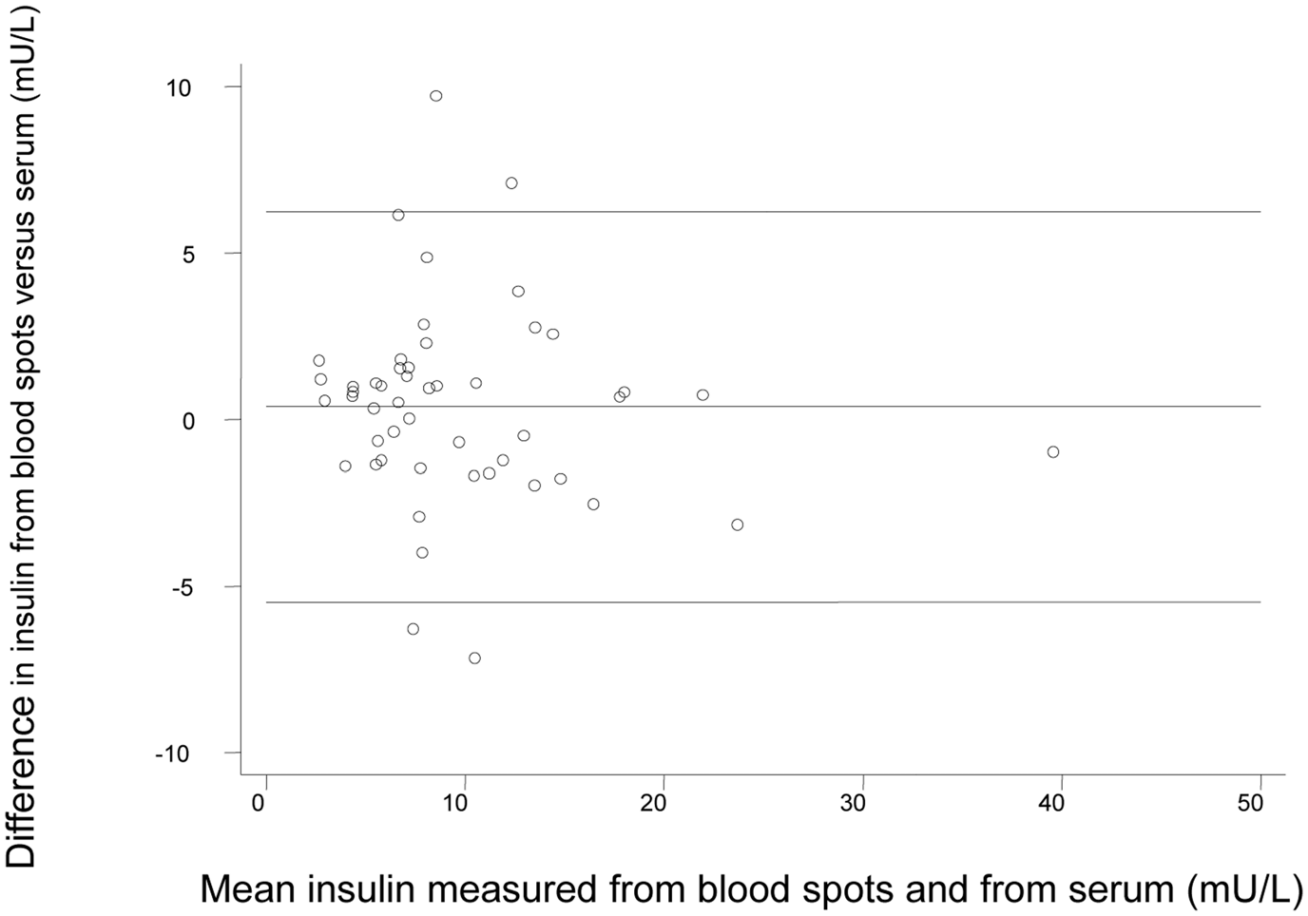
Bland-Altman plot for fasted bloodspot versus serum insulin assay in 50 paired samples.

### Inter- and Intra-assay Imprecision

Inter- and intra-assay imprecision was assessed using internal quality control samples at two levels: low (6.7 mU/L) and high (23.1 mU/L). Intra-assay imprecision for low and high concentrations of insulin was established from duplicate analysis (n = 157). The inter-assay imprecision was determined from 33 separate runs over a period of 15 months. Both intra- and inter-assay imprecision include between-spot extraction variations.

**Table 2:** Geometric mean (log standard deviation) insulin levels in boys and girls and association with demographic and clinical characteristics, N = 12,812.

	Geometric mean (log SD) insulin levels (mU/L) and age-adjusted difference or change in insulin (95% CI) per unit of demographic/clinical variable				
	N	Girls	N	Boys	p for sex interaction
**Age group (yrs)**					
10–<11	385	3.1 (1.0)	393	2.3 (1.1)	
11–<12	4851	4.2 (1.0)	4955	3.1 (1.1)	
12–<13	962	3.8 (1.1)	1049	2.9 (1.2)	
13–<14	100	1.7 (1.6)	117	2.3 (1.5)	0.01
**Location**					
Rural	2654	4.0(1.0)	2694	2.9(1.1)	
Urban	3644	4.0(1.0)	3820	3.0(1.1)	
*Difference in insulin* a	6298	1% (−19%, 26%)	6514	3% (−18%, 29%)	0.37
East	3022	4.1(1.0)	3004	3.0(1.1)	
West	3276	3.9(1.1)	3510	2.9(1.1)	
*Difference in insulin* a	6298	−3% (−23%, 22%)	6514	−3% (−23%, 22%)	0.87
**Tanner stage by breast (girls)/genitalia (boys)**					
I	705	2.7(1.0)	872	2.9(1.0)	
II	3241	3.8(1.0)	3515	2.9(1.1)	
III	2029	4.7(1.0)	1881	3.1(1.2)	
IV/V	318	5.4(1.0)	238	3.4(1.3)	
*Change in insulin per category* a	6293	30% (21%, 39%)	6506	4% (−7%, 16%)	<0.001
**Tanner stage by pubic hair assessment**					
I	1751	3.2(1.0)	3248	2.8(1.1)	
II	2962	4.1(1.0)	2684	3.1(1.1)	
III	1402	4.9(1.0)	525	3.7(1.2)	
IV/V	178	6.0(1.0)	49	4.5(1.0)	
*Change in insulin per category* a	6293	28% (19%, 37%)	6506	15% (5%, 26%)	0.01
**Body mass index (kg/m^2^)** b					
Normal weight	5432	3.7(1.0)	5454	2.7(1.1)	
Overweight	734	6.6(0.9)	819	4.5(1.0)	
Obese	108	9.3(0.8)	213	6.5(0.8)	
*Change in insulin per category* a	6274	70% (58%, 84%)	6486	60% (51%, 69%)	0.93
**Waist circumference (cm)**					
<90^th^ centile	5689	3.7(1.0)	5811	2.7(1.1)	
≥90^th^ centile	595	7.7(0.8)	679	5.6(1.0)	
*Difference in insulin* a	6284	108% (87%, 130%)	6490	105% (84%, 128%)	0.68
**Fat mass (kg)**					
<90^th^ centile	5410	3.7(1.0)	6005	2.8(1.1)	
≥90^th^ centile	798	6.7(1.0)	449	6.2(0.9)	
*Difference in insulin* a	6208	83% (64%, 105%)	6454	121% (91%, 157%)	0.03
**Triceps thickness (mm)**					
<90^th^ centile	5658	3.8(1.0)	5963	2.8(1.1)	
≥90^th^ centile	628	6.8(0.9)	533	5.4(1.0)	
*Difference in insulin* a	6286	82% (56%, 112%)	6496	93% (70%, 119%)	0.85
**Subscapular thickness (mm)**					
<90^th^ centile	5621	3.7(1.0)	5926	2.8(1.1)	
≥90^th^ centile	627	6.9(0.9)	532	5.3(1.0)	
*Difference in insulin* a	6248	84% (65%, 105%)	6458	91% (74%, 110%)	0.68
**Height (cm)**					
Q1	1412	2.9(1.0)	1793	2.3(1.1)	
Q2	1628	3.7(1.0)	2006	3.0(1.1)	
Q3	1490	4.4(1.0)	1448	3.2(1.1)	
Q4	1766	5.1(1.0)	1263	4.0(1.1)	
*Change in insulin per 10 cm* a	6296	36% (30%, 43%)	6510	33% (25%, 42%)	0.80
**Birthweight (g)**					
Q1	1974	4.0(1.0)	1369	2.9(1.2)	
Q2	1660	4.0(1.1)	1430	3.0(1.1)	
Q3	1485	3.9(1.1)	1796	3.0(1.1)	
Q4	1179	4.1(1.0)	1916	3.0(1.1)	
*Change in insulin per 100g* a	6298	0% (−1%, 1%)	6511	0% (−1%, 1%)	0.55
**Glucose(mmol/l)**					
Q1	2069	3.4(1.1)	2053	2.4(1.2)	
Q2	1246	4.0(1.0)	1307	3.0(1.0)	
Q3	1449	4.5(1.0)	1526	3.3(1.1)	
Q4	1525	4.5(1.0)	1623	3.5(1.1)	
*Change in insulin per SD increase in glucose* a	6289	12% (5%, 18%)	6509	19% (12%, 25%)	0.19

SD = standard deviation; CI = confidence interval.

a Linear regression coefficient and 95% CI (age-adjusted).

b Normal weight, overweight and obese as defined by the International Obesity Task Force, IOTF BMI cut-offs. [40].

### Stability

Stability studies were conducted at both the *Mercodia* laboratory and the central analytical laboratory for the PROBIT trial based in Minsk. *Mercodia* conducted a short-term stability study in which 16 sets of bloodspot insulin standards were stored at −80°C for up to 2 months and exposed to up to four freeze–thaw cycles. *Mercodia* also assessed the stability of the samples when stressed at +30°C for one hour, 24 hours, 48 hours and one week. To assess stability at −80°C over the longer term, 3 sets of whole blood samples in Minsk were spotted onto Whatman 903 cards, cut into strips containing one blood spot per strip, and frozen at −80°C. Prior to freezing, baseline (pre-storage) values were established using 30 replicates of each fresh blood spot sample. Approximately monthly, for a period of 26 months, a different strip was thawed (to ensure insulin levels were based on only one thaw) and the insulin value measured. We also stored low (6.7 mU/L) and high (23.1 mU/L) internal quality control samples, prepared on the same occasion in the same batch by Mercodia, at −80°C and repeated the assay at regular intervals, after between 7 and 31 months of frozen storage (the IQCs were stored for longer than the Minsk samples).

### Comparison between Sera and Dried Blood Spots

We compared fasting insulin concentrations in serum (measured using Mercodia’s Human Insulin ELISA) with insulin concentrations measured from dried blood spots, collected simultaneously from 50 children in Minsk. Whole blood samples were allowed to stand at room temperature for 2–4 hours then centrifuged at 3000 rpm for 5 minutes before separating the serum. Blood spots and sera were stored at −80°C and brought to room temperature before assaying.

### Distribution of Insulin by Demographic and Clinical Characteristics

We followed up 13,879 children aged 11.5 years (interquartile range 11.3–11.8 years) who were participants in a multicentre trial of a breastfeeding promotion intervention involving 31 polyclinics (39 paediatricians) located throughout the Republic of Belarus. [34] Children who were eligible for the trial (healthy, term newborns whose mother initiated breastfeeding) were originally recruited at birth between 1996 and 1997, [34] and have been followed up intermittently since then, including the 11.5 year follow-up reported here between 2008–2010. At 11.5 years, dried blood spots were collected from the children as described below. The children also had whole blood fasting glucose measured by glucometer (Roche ACCU-CHEK Advantage meter system, F. Hoffmann-La Roche AG, Basel, Switzerland) and the following physical measurements (amongst others not reported here) were made by study paediatricians: Tanner pubertal stage, height, weight, body mass index (BMI, weight (kg)/height (m)^2^), skinfold thicknesses, waist circumference and leg-to-leg bioimpedence (Tanita TBF 300GS, Tanita Europe BV, Netherlands) for calculation of fat-mass. Birthweight had been collected when the children were recruited into the trial at birth in 1996/1997. [34].

### Sample Collection

Children participating in the PROBIT trial follow-up were asked to fast for at least 8 hours. Up to 8 drops of fingertip capillary blood were obtained by paediatricians and applied to the pre-printed circles to form one discrete spot per drop, following specific guidelines [35], [39] and training (see below). The fingerprick was made using a sterile, single-use disposable lancet device with three depth settings designed to minimize pain (Roche ACCU-CHEK Safe-T-Pro Plus lancet, Roche Diagnostics Corp., Indianapolis, USA). To avoid contact of the fresh blood filter-paper sample with potential contaminants, the sample was air-dried on bespoke drying racks for 3–4 hours and the dried blood spots placed in low gas-permeable zip-closure plastic bags with desiccant packages. [36] These were stored initially in –20°C freezers at the 31 polyclinics, prior to transfer in cool boxes packed with freezer packs to −80°C freezers at the central laboratory in Minsk. The samples were stored at −20°C for a median of 1.6 months (IQR 1.0–5.0 months) and at −80°C for a median of 16.9 (IQR 12.6–20.2) months.

### Training in Blood Spot Sampling

Paediatricians received training at a 1-day workshop and were provided with a written manual of procedures and an illustrative DVD. [35], [39] Training included: the importance of proper technique for ensuring adequate blood flow (warm fingers, held below lap); emphasizing the need to allow blood to well on the finger-tip (rather than apply excessive pressure) to minimize haemolysis or dilution of the sample by interstitial fluid; and the need to allow large drops of blood to pool on the finger before dropping them once onto the pre-printed filter circles (with no blotting or re-spotting) to ensure adequate and evenly distributed sample volume. Training was conducted by a consultant biochemist (Dr Gusina) with extensive experience of running a national neonatal screening programme based on filter-paper blood spot collections. Feedback on the quality of spots was provided to the paediatricians at workshops every 6-months during the study fieldwork period. As blood spot cards arrived at the central laboratory in Minsk, laboratory staff assessed and recorded the quantity and quality of blood spots. Any paediatrician noted to have consistent problems (e.g. blotting, spotting, fewer than 6 good spots on average) was contacted by Dr Gusina to discuss techniques to improve the quality of the blood spot collection.

### Ethics

The study received ethical approval from McGill University Health Centre Research Ethics Board; the Human Subjects Committees at Harvard Pilgrim Health Care; and the Avon Longitudinal Study of Parents and Children Law and Ethics Committee. The conduct of the study was also approved by the Ministry of Health of the Republic of Belarus. A parent or legal guardian provided written informed consent and all children provided written assent. The trial registration number for Current Controlled Trials is ISRCTN37687716.

### Statistical Analysis

Calibration curves were constructed with Multicalc software (Wallac, Turku, Finland), using the log scale for both x and y axes. To assess the relationship between insulin values from serum versus blood spot samples collected simultaneously, we computed the intraclass correlation coefficient (95% confidence interval) using one-way analysis of variance and constructed a Bland-Altman plot of the difference between the serum and bloodspot insulin values (y-axis) against the mean of these two values (x-axis). Linear regression accounting for clustering by polyclinic was used to investigate relationships of fasting insulin levels with storage time in the −80°C freezer, age, location of polyclinic (urban/rural; East/West Belarus), puberty as measured by Tanner stage, measures of general (BMI, fat mass), peripheral (triceps skinfold thickness) and central (subscapular skinfold thickness, waist circumference) adiposity, height, birthweight and fasting glucose. We calculated sex-specific associations and performed a likelihood ratio test for interactions between sex and the demographic, glucose and anthropometric factors on insulin. Insulin concentrations were log-transformed (ln(x)) for the analyses and geometric means and log standard deviations are presented. Insulin values below the lowest standard (1 mU/L, n = 1,458) were assigned the observed value (mean = 0.5 mU/L, IQR = 0.3–0.7), after determining amongst a sample of 80 children that repeating the assay for these low values made no material difference to the observed values. A sensitivity analysis excluding those 1,458 children with an insulin concentration below 1 mU/L did not alter the observed associations of insulin with demographic and clinical variables. All analyses were undertaken using Stata version 11 (StataCorp LP, Texas).

## Results

A typical calibration curve is shown in **Figure 1** **.** The precision of the dried blood spot extraction and consecutive determination by ELISA is summarised in **Table 1**. Mean intra-assay (n = 157) coefficients of variation were 15% and 6% for ‘low’ (6.7 mU/L) and ‘high’ (23.1 mU/L) values, respectively; the respective inter-assay values (n = 33) were 23% and 11%.

**Figure 2** shows a comparison of insulin values for 16 dried blood spot samples stored at −80°C for 8 weeks versus insulin concentrations measured in dried blood spots prior to frozen storage (mean: 9.1 mU/L; range: 1.7 mU/L to 23.2 mU/L). These data show a very strong correlation between insulin levels measured before (fresh sample), compared to those measured after, freezing (intraclass correlation coefficient = 0.99; 95% confidence interval, CI: 0.99 to 1.00). The mean difference between values obtained from fresh versus frozen samples was very small and the 95% confidence interval was consistent with no true difference (0.2 mU/L; 95% CI: 0.0 to 0.5). A Bland-Altman plot suggested that low values of insulin were slightly increased, and high values slightly reduced, after 8 weeks frozen storage (**Figure 3**). However, the differences were small and the 95% limits of agreement were within 1 mU/L. When three separate samples were stored at +30°C, the values at 1 hour, 24 hours, 48 hours and 1 week were between 89% and 127% of the values at time 0 (**Figure 4**).

The results of the longer-term stability study at −80°C are shown in **Figures 5** **and** **6**. Most values were within the 95% reference range established from 30 replicates of the time 0 value. In linear regression analyses, there was no evidence of any association between storage time at −80°C and levels of insulin for sample 1 (percentage change in insulin levels per month = −0.17%; 95% CI: −1.15 to 0.80; p = 0.72), sample 2 (0.12%; 95% CI: −0.62 to 0.87; p = 0.74), and the low (−0.35%; 95% CI: −1.42 to 0.73; p = 0.51) and high (0.09%; 95% CI: −0.71 to 0.89; p = 0.81) internal quality control samples. There were month-by-month fluctuations in line with the inter-assay imprecision of measurements and in sample 3, the 18, 19 and 22 month readings were lower than expected. Overall sample 3 was stable over 26 months, however, with a mean change in insulin levels per month of −2.19%; (95% CI: −4.79 to 0.41; p = 0.10). **Figure 7** shows that insulin concentrations for 3 samples remained stable to being subjected to 4 freeze-thaw cycles.

### Comparison of Samples

Comparison of 50 paired fasted whole bloodspots and serum samples collected simultaneously gave an intraclass correlation coefficient of 0.90 (95% confidence interval: 0.85 to 0.95) (**Figure 8** **)**, the range of serum values being 3.2 to 39.1 mU/L. The Bland Altman plot shows that the 95% limits of agreement were between −5.5 to +6.2 mU/L with a mean difference across the range of values of 0.39 mU/L (CI −0.45 to 1.22) and no evidence of a difference in variance across mean values (p = 0.41) (**Figure 9**).

### Associations of Insulin with Demographic and Clinical Variables

Out of a total of 13,879 children who were approached, a total of 13,487 (97.2%) provided at least one and 13,403(96.6%) provided at least two acceptable dried blood spot samples (two were sufficient for the insulin assay). Amongst the 13,487 children, the paediatricians collected a median of 8 blood spots, with 84.1% (11,347) of children assessed by the central laboratory in Minsk as having had 8 acceptable quality blood spots collected. We assayed a total of 12,812 children who fasted for at least 8 hours. **Table 2** shows mean (log standard deviation) insulin levels in boys and girls by the following variables: age-group; location of the polyclinic (urban/rural and East/West); Tanner stage; BMI (normal weight, overweight and obese as defined by the International Obesity Task Force, IOTF BMI cut-offs [40]); waist circumference, fat mass, triceps and subscapular thicknesses (above or below the 90th centile); and quartiles of fasting glucose, height and birthweight. In both boys and girls, mean insulin levels increased with Tanner stage by pubic hair; all measures of adiposity (BMI, waist circumference, fat mass and skinfold thicknesses); concurrent glucose levels; and height. In girls, insulin levels were also positively related to Tanner stage by breast development.

## Discussion

The advantages of fingerstick blood collection onto filter paper include ease of processing, storage and transport, thus providing a valuable research tool for large epidemiology studies, particularly those that are geographically dispersed. We have shown that insulin in dried blood spots is stable and can be reliably quantified on a very large-scale with modest infrastructure, making the method useful for population-based studies of insulin resistance, diabetes and cardiovascular risk in a variety of settings.

The dried blood spot methods showed acceptable analytical performance characteristics and good agreement with the conventional serum insulin assay across the distribution of insulin values. There are several sources of variation unique to bloodspot sampling and the assay that could explain differences between bloodspot versus serum. For example, proper placement of the whole blood sample on the filter paper is critical and errors can be introduced if blood is blotted or smeared rather than drawn onto the filter paper by capillary action. [32] We minimised this source of error by rigorous training and regular feedback. Pre-printed circles on the filter paper were used to help guide the paediatricians to position the bloodspot. To maximise stability, samples were placed in zip-closure bags packed with dessicant and frozen at −20°C promptly after drying to reduce the chances of degradation, although our stability study suggested insulin was stable in dried blood spots when exposed to +30°C for one week. These sources of variation are not seen with venepuncture sampling. The haematocrit level was set to 0.4 in the standards but not adjusted for in each sample; any sample differences would introduce errors. The exact reason why there were outliers outside the 95% limits of agreement in **Figure 9** is not clear, but may be related to the quality of the blood spots or haematocrit variation. The variation seen in **Figures 5** **and** **6** is broadly in line with the imprecision suggested in **Table 1**, although in **Figure 6**, the CV for the high IQC samples used in the stability study was slightly higher at 18%, which could reflect sources of variation outlined above. Despite unique sources of error, we achieved a good correlation between dried blood spot versus serum methods of measurement. In addition, although bloodspot assays are inherently less precise than serum assays, the present ELISA method exhibited intra- and inter-assay coefficients of variation (see **Table 1**) that compare favourably with those reported for radioimmunoassay (14.0% and 25.0%, respectively) [32] and chemiluminescence (11.7–29.8% and 9.8–15.5%, respectively, from Table 3 of reference [33]) methods for insulin quantification in dried blood spots.

This is the first study we are aware of to report the long-term stability of insulin in dried blood spots, and our data show stability for over 2 years when frozen at −80°C. Reassuringly, we have shown that samples can be left at very high room temperatures for up to a week (+30°C) and remain stable, providing some flexibility when samples are being collected in the field (e.g. in remote sites with limited immediate access to freezers).

A range of 1–50 mU/L was selected for the assay to cover the anticipated whole blood insulin concentrations of children. Our lower limit of detection (1 mU/L) and high intraclass correlation (0.90) between serum and dried blood spot measurements, collected simultaneously, compares favourably with the chemiluminescent immunoassay assay developed by Butter *et al* (lower limit of detection = 0.8 mU/L; correlation = 0.89 between paired whole bloodspots and serum samples). [33] The close agreement between dried blood spot (measured against bloodspot standards) and conventional serum (measured against liquid buffer standards) assays strongly supports the validity of dried bloodspot insulin analysis. We have also demonstrated that insulin is stable when exposed to at least four freeze-thaw cycles, indicating that samples can be reanalysed without loss of insulin.

Red blood cells contain an insulin degrading enzyme that cleaves insulin. We did not investigate the effect of adding an insulin degrading enzyme inhibitor to the eluate, because Butter et al showed that this did not improve insulin concentrations measured from whole bloodspots, [33] speculating that since the action of applying whole blood to filter paper causes red blood cells to lyse, this may release insulin degrading enzymes before assay incubation.

In our fieldwork study, we showed that taking capillary blood samples from children is feasible on a large scale, since only 3% (n = 392) of 13,879 children approached did not provide a useable sample and the paediatricians were able to take a median of 8 acceptable blood spots amongst those who were sampled. Although we documented slightly greater imprecision than would be expected in serum samples, the mean values in girls and boys that we observed, and our analysis of established associations of insulin levels with adiposity measures, puberty, glucose and height, were in line with previously published results. [41].

The advantages of filter paper capillary blood sampling, combined with the acceptable analytical performance characteristics of the dried blood spot assays, makes the approach ideal for large-scale epidemiologic applications, including studies of children, whenever the acceptability, stability, cost and safety of liquid sample collection and transportation to distant laboratories may be limiting factors. There are caveats, as previously pointed out, including the need for adequate training for those taking the bloodspot samples onto filter paper, to avoid several sources of error, and the fact that we have developed the dried bloodspot assay for research and not clinical use, which would require additional investigation. [42].

## References

[1] Reaven GM (1995) Pathophysiology of insulin resistance in human disease. Physiological Reviews 75: 473–486. 7624391 10.1152/physrev.1995.75.3.473

[2] Srinivasan SR, Myers L, Berenson GS (2002) Predictability of childhood adiposity and insulin for developing insulin resistance syndrome (syndrome X) in young adulthood: the Bogalusa Heart Study. Diabetes 51: 204–209. 11756342 10.2337/diabetes.51.1.204

[3] Cook S, Weitzman M, Auinger P, Nguyen M, Dietz WH (2003) Prevalence of a Metabolic Syndrome Phenotype in Adolescents: Findings From the Third National Health and Nutrition Examination Survey, 1988–1994. Archives of Pediatrics Adolescent Medicine 157: 821–827. 12912790 10.1001/archpedi.157.8.821

[4] Dwyer T, Blizzard L, Venn A, Stankovich JM, Ponsonby AL, et al (2002) Syndrome X in 8-y-old Australian children: stronger associations with current body fatness than with infant size or growth. International Journal of Obesity & Related Metabolic Disorders: Journal of the International Association for the Study of Obesity 26: 1301–1309. 10.1038/sj.ijo.0802111 12355325

[5] Cruz ML, Huang TTK, Johnson MS, Gower BA, Goran MI (2002) Insulin Sensitivity and Blood Pressure in Black and White Children. Hypertension 40: 18–22. 12105132 10.1161/01.hyp.0000019972.37690.ef

[6] Sinha R, Fisch G, Teague B, Tamborlane WV, Banyas B, et al (2002) Prevalence of Impaired Glucose Tolerance among Children and Adolescents with Marked Obesity. The New England Journal of Medicine 346: 802–810. 11893791 10.1056/NEJMoa012578

[7] McGill HC Jr, McMahan CA, Herderick EE, Zieske AW, Malcom GT, et al (2002) Obesity Accelerates the Progression of Coronary Atherosclerosis in Young Men. Circulation 105: 2712–2718. 12057983 10.1161/01.cir.0000018121.67607.ce

[8] Bao W, Srinivasan SR, Berenson GS (1996) Persistent elevation of plasma insulin levels is associated with increased cardiovascular risk in children and young adults. The Bogalusa Heart Study. Circulation 93: 54–59. 8616941 10.1161/01.cir.93.1.54

[9] Bao W, Srinivasan SR, Wattigney WA, Berenson GS (1994) Persistence of multiple cardiovascular risk clustering related to syndrome X from childhood to young adulthood. The Bogalusa Heart Study. Arch Int Med 154: 1842–1847. 8053753

[10] Weyer C, Bogardus C, Mott DM, Pratley RE (1999) The natural history of insulin secretory dysfunction and insulin resistance in the pathogenesis of type 2 diabetes mellitus. J Clin Invest 104: 787–794. 10491414 10.1172/JCI7231 PMC408438

[11] Hanson RL, Imperatore G, Bennett PH, Knowler WC (2002) Components of the “Metabolic Syndrome” and Incidence of Type 2 Diabetes. Diabetes 51: 3120–3127. 12351457 10.2337/diabetes.51.10.3120

[12] Laaksonen DE, Lakka HM, Niskanen LK, Kaplan GA, Salonen JT, et al (2002) Metabolic syndrome and development of diabetes mellitus: application and validation of recently suggested definitions of the metabolic syndrome in a prospective cohort study. Am J Epidemiol 156: 1070–1077. 12446265 10.1093/aje/kwf145

[13] Pyorala M, Miettinen H, Halonen P, Laakso M, Pyorala K (2000) Insulin Resistance Syndrome Predicts the Risk of Coronary Heart Disease and Stroke in Healthy Middle-Aged Men : The 22-Year Follow-Up Results of the Helsinki Policemen Study. Arteriosclerosis, Thrombosis, and Vascular Biology 20: 538–544. 10.1161/01.atv.20.2.538 10669654

[14] Lempiainen P, Mykkanen L, Pyorala K, Laakso M, Kuusisto J (1999) Insulin Resistance Syndrome Predicts Coronary Heart Disease Events in Elderly Nondiabetic Men. Circulation 100: 123–128. 10402440 10.1161/01.cir.100.2.123

[15] Hanley AJG, Williams K, Stern MP, Haffner SM (2002) Homeostasis Model Assessment of Insulin Resistance in Relation to the Incidence of Cardiovascular Disease: The San Antonio Heart Study. Diabetes Care 25: 1177–1184. 12087016 10.2337/diacare.25.7.1177

[16] Isomaa B, Almgren P, Tuomi T, Forsen B, Lahti K, et al (2001) Cardiovascular Morbidity and Mortality Associated With the Metabolic Syndrome. Diabetes Care 24: 683–689. 11315831 10.2337/diacare.24.4.683

[17] Lakka HM, Laaksonen DE, Lakka TA, Niskanen LK, Kumpusalo E, et al (2002) The metabolic syndrome and total and cardiovascular disease mortality in middle-aged men. JAMA 288: 2709–2716. 12460094 10.1001/jama.288.21.2709

[18] Kuh D, Ben-Shlomo Y (1997) A life course approach to chronic disease epidemiology. Oxford: OUP. 11980781

[19] Barker DJP (1998) Mothers, babies and health in later life. London: Churchill Livingstone.

[20] Singhal A, Lucas A (2004) Early origins of cardiovascular disease: is there a unifying hypothesis? Lancet 363: 1642–1645. 15145640 10.1016/S0140-6736(04)16210-7

[21] Lawlor DA, Ebrahim S, Davey Smith G (2002) British women’s heart and health study (2002) Socioeconomic position in childhood and adulthood and insulin resistance: cross sectional survey using data from British women’s heart and health study. BMJ 325: 805–807. 12376440 10.1136/bmj.325.7368.805 PMC128946

[22] Davey Smith G, Hart C (1997) Insulin resistance syndrome and childhood social conditions. Lancet 349: 284–285. 10.1016/s0140-6736(05)64894-5 9014935

[23] Davey Smith G, Greenwood R, Gunnell D, Sweetnam P, Yarnell J, et al (2001) Leg length, insulin resistance, and coronary heart disease risk: the Caerphilly Study. J Epidemiol Community Health 55: 867–872. 11707479 10.1136/jech.55.12.867 PMC1731819

[24] Lawlor DA, Davey SG, Ebrahim S (2002) Birth weight of offspring and insulin resistance in late adulthood: cross sectional survey. British Medical Journal 325: 359. 12183306 10.1136/bmj.325.7360.359 PMC117884

[25] Singhal A, Fewtrell M, Cole TJ, Lucas A (2003) Low nutrient intake and early growth for later insulin resistance in adolescents born preterm. Lancet 361: 1089–1097. 12672313 10.1016/S0140-6736(03)12895-4

[26] Ravelli ACJ, van der Meulen JH, Osmond C, Barker DJP, Bleker OP (2000) Infant feeding and adult glucose tolerance, lipid profile, blood pressure, and obesity. Arch Dis Child 82: 248–252. 10685933 10.1136/adc.82.3.248 PMC1718232

[27] Rung J, Cauchi S, Albrechtsen A, Shen L, Rocheleau G, et al (2009) Genetic variant near IRS1 is associated with type 2 diabetes, insulin resistance and hyperinsulinemia. Nature Genetics 41: 1110–1115. 19734900 10.1038/ng.443

[28] Expert Panel on Detection Evaluation, Treatment of High Blood Cholesterol in Adults (2001) Executive Summary of the Third Report of the National Cholesterol Education Program (NCEP) Expert Panel on Detection, Evaluation, and Treatment of High Blood Cholesterol in Adults (Adult Treatment Panel III). JAMA 285: 2486–2497. 11368702 10.1001/jama.285.19.2486

[29] Parker SP, Cubitt WD (1999) The use of the dried blood spot sample in epidemiological studies. J Clin Pathol 52: 633–639. 10655983 10.1136/jcp.52.9.633 PMC501537

[30] Lynch JF, Marshall MD, Wang XL, Wilcken DE (1998) Apolipoprotein screening in Australian children: feasibility and the effect of age, sex, and ethnicity. Medical Journal of Australia 168: 61–64. 9469184 10.5694/j.1326-5377.1998.tb126712.x

[31] Worthman CM, Stallings JF (1997) Hormone measures in finger-prick blood spot samples: new field methods for reproductive endocrinology. American Journal of Physical Anthropology 104: 1–21. 9331450 10.1002/(SICI)1096-8644(199709)104:1<1::AID-AJPA1>3.0.CO;2-V

[32] McDade TW, Williams S, Snodgrass JJ (2007) What a drop can do: dried blood spots as a minimally invasive method for integrating biomarkers into population-based research. Demography 44: 899–925. 18232218 10.1353/dem.2007.0038

[33] Butter NL, Hattersley AT, Clark PM (2001) Development of a bloodspot assay for insulin. Clinica Chimica Acta 310: 141–150. 10.1016/s0009-8981(01)00545-9 11498079

[34] Kramer MS, Chalmers B, Hodnett ED, Sevkovskaya Z, Dzikovich I, et al (2001) Promotion of Breastfeeding Intervention Trial (PROBIT): a randomized trial in the Republic of Belarus. JAMA 285: 413–420. 11242425 10.1001/jama.285.4.413

[35] Mei JV, Alexander JR, Adam BW, Hannon WH (2001) Use of Filter Paper for the Collection and Analysis of Human Whole Blood Specimens. Journal of Nutrition 131: 1631S–1636. 11340130 10.1093/jn/131.5.1631S

[36] National Committee for Clinical Laboratory Standards (NCCLS) (1997) Blood Collection on Filter Paper for Neonatal Screening Programs, approved standard, National Committee for Clinical Laboratory Standards Document A4A3 3rd edition. Wayne, PA: National Committee for Clinical Laboratory Standards.

[37] Miller WG, Thienpont LM, Van Uytfanghe K, Clark PM, Lindstedt P, et al (2009) Toward Standardization of Insulin Immunoassays. Clinical Chemistry 55: 1011–1018. 19325009 10.1373/clinchem.2008.118380

[38] International Organisation for Standardisation (2003) ISO 11843-4: 2003. Capability of detection - Part 4: Methodology for comparing the minimum detectable value with a given value. Geneva: International Organisation for Standardisation.

[39] Warnick GR, Leary ET, Ammirati EB, Allen MP (1994) Cholesterol in fingerstick capillary specimens can be equivalent to conventional venous measurements. Archives of Pathology & Laboratory Medicine 118: 1110–1114. 7979896

[40] Cole TJ, Bellizzi MC, Flegal KM, Dietz WH (2000) Establishing a standard definition for child overweight and obesity worldwide: international survey. BMJ 320: 1240–1243. 10797032 10.1136/bmj.320.7244.1240 PMC27365

[41] Moran A, Jacobs DR, Steinberger J, Hong CP, Prineas R, et al (1999) Insulin resistance during puberty: results from clamp studies in 357 children. Diabetes 48: 2039–2044. 10512371 10.2337/diabetes.48.10.2039

[42] Williams SR, McDade TW (2009) The Use of Dried Blood Spot Sampling in the National Social Life, Health, and Aging Project. J GERONTOL B PSYCHOL SCI SOC SCI gbn022. 10.1093/geronb/gbn022 PMC2763524 19244547

